# Role of ω3 polyunsaturated fatty acids in diabetic retinopathy: a morphological and metabolically cross talk among blood retina barriers damage, autoimmunity and chronic inflammation

**DOI:** 10.1186/s12944-019-1049-9

**Published:** 2019-05-15

**Authors:** Aldo R. Eynard, Gaston Repossi

**Affiliations:** Instituto de Biología Celular, Histología y Embriología, Facultad de Ciencias Médicas, INICSA (CONICET-Universidad Nacional de Córdoba), Córdoba, Argentina

**Keywords:** Diabetic retinopathy, Inflammation, Autoimmunity, Rod and cones, Polyunsaturated fatty acids, Elderly

## Abstract

Vision disorders are one of the most serious complications of diabetes mellitus (DM) affecting the quality of life of patients and eventually cause blindness. The ocular lesions in diabetes mellitus are located mainly in the blood vessels and retina layers. Different retina lesions could be grouped under the umbrella term of diabetic retinopathies (DMRP).

We propose that one of the main causes in the etiopathogenesis of the DMRP consists of a progressive loss of the selective permeability of blood retinal barriers (BRB). The loss of selective permeability of blood retinal barriers will cause a progressive autoimmune process. Prolonged autoimmune injures in the retinal territory will triggers and maintains a low-grade chronic inflammation process, microvascular alterations, glial proliferation and subsequent fibrosis and worse, progressive apoptosis of the photoreceptor neurons.

Patients with long-standing DM disturbances in retinal BRBs suffer of alterations in the enzymatic pathways of polyunsaturated fatty acids (PUFAs), increase release of free radicals and pro-inflammatory molecules and subsequently incremented levels of vascular endothelial growth factor. These facts can produce retinal edema and photoreceptor apoptosis.

Experimental, clinical and epidemiological evidences showing that adequate metabolic and alimentary controls and constant practices of healthy life may avoid, retard or make less severe the appearance of DMRP. Considering the high demand for PUFAs ω3 by photoreceptor complexes of the retina, it seems advisable to take fish oil supplements (2 g per day). The cellular, subcellular and molecular basis of the propositions exposed above is developed in this article.

Synthesizer drawings the most relevant findings of the ultrastructural pathology, as well as the main metabolic pathways of the PUFAs involved in balance and disbalanced conditions are provided.

## Introduction

Diabetes Mellitus (DM) involving progressive alterations in metabolic and inflammatory indices comprising perturbations of the metabolism of glucose, lipids and proteins. Increases in the oxidative stress and alterations in glucose metabolism result in elevation of some inflammatory markers as leukotrienes (LTB4), interleukins-2 and 6 (IL-2 and IL-6), activation of toll-like receptor 4 and C reactive protein [1–3].

The global increasing incidence of DM not only impacts the health of the affected individual but also enhances the cost of health care thus having implications for political, economic and social concerns for the society mainly in long standing aged diabetic patients [4, 5]. DM is estimated to affect about 422 million people in the world actually and 1.6 million deaths worldwide were directly caused by diabetes in 2016 [6].

DM seems to be closely linked to scarce physical activity and inappropriate food intake resulting in obesity, insulin resistance, and eventually in metabolic syndrome [7]. One of the latest severe complication of DM in long standing patients is partial or total blindness preceded by others visual perturbations englobed in this article under the term DM-linked retinopathy (DMRP) [8, 9].

Increasing number of reports coincidently point out that a disbalance in the metabolism of ω3, ω6 and ω9 polyunsaturated fatty acids (PUFAs) occurs in obesity, insulin resistance, metabolic syndrome and DM [1, 10, 11]. In this regards it is comparatively less known the role played by disturbed PUFA metabolism in the DMRP-affected eye [8, 12]. The role of disbalanced metabolism of ω3 and ω6 PUFAs and their metabolites as lipoxins, resolvins, protectins and maresins in the development of a low grade chronic inflammation (LGCI) and its impact on the pathophysiology of DMRP have comprehensively been revised [13–15].

Plasma membranes (PM) of highly differentiated sacs and vesicles of retinal cones and rods of outer segment (OS) are unusually rich in long chain highly unsaturated-PUFAs (LCHU-PUFA) mainly ω3-docohexaenoic (DHA) and ω3-eicosapentaenoic (EPA) and ω6-arachidonic (AA) [16, 17]. Phospholipids (PL) containing these LCHU-PUFAs are heavily concentrated in PMs of OS which are in risk of becoming abnormal because relative deficiency of ω3 LCHU-PUFAs [18, 19].

Human retina is a privileged and “sequestrated” highly differentiated neural cell populations without further contacts with the own immunological system (IS) of each individual [20, 21] precluding a significative role for autoimmune responses in the pathophysiology of DMRP. This possibility have been earlier proposed by Rahi and Addison in 1983 [22] and others whom critically discussed cardinal findings in DMRP [21]. Frequent abnormalities in retinal tissues of DM subjects comprise higher levels of anti-pericyte and anti-endothelial cell autoantibodies, increased levels of tumor necrosis factor-alpha (TNF-α), several pro-inflammatory interleukins and lymphokines in the serum and vitreous, increased deposits of immunoglobulins in pre-retinal membranes and activation of complement system [23]. DM subjects showed higher values of several leukocyte antigens and their receptors on retinal blood vessels and pigmented epithelial cells (PEC). Described elevated levels of autoantigens against retinal epitopes and increased expression of their receptors observed on DMRP are similar to those registered in many other nonlymphoid cells populations in several autoimmune diseases [24], including type 1 DM [25]. Hence this abnormal expression of neo-antigens in retinal neuron cells seems to be part of a progressive autoimmune response [26].

The role played by abnormal metabolism of PUFAs ω3 and ω6 and their metabolites and their impact on development of LGCI on DMRP have been discussed [8, 27, 28]. In this review the aim is highlight the morphological-linked perturbations in retina as a whole, and particularly those changes observed in the inner and outer blood retina barrier (BRB) and photoreceptors, in the framework of a functional/ metabolically deficiency of ω3 LCHU-PUFAs, topics scarcely integrated in these issues.

### Human retina

#### Histogenesis and neural cell populations in human retina

The histogenesis of the human neural retina involves complex genetic and epigenetic sequential processes controlled by on/off switching of genes groups modulating waves of neuroblasts proliferation, differentiation, migration, selective apoptosis and angiogenesis being some of these stages bizarrely reactivated in DMRP [29, 30].

Genetic planification and expression of human retina development involve several progenitor genes (such as LIN28B, FGF19, PRTG, and SFRP2) [31]. Interestingly, SFRP2 overexpression has been linked to obesity, insulin resistance and increased vascular endothelial growth factor (VEGF) [32]. Since human retina is a prolongation of the central nervous system (CNS) its highly differentiated neural cell populations and their bulk of expressed new molecules (potential epitopes) will remain sequestrated within their own compartments and without further contacts with the still maturating IS of each individual [20, 21, 33]. Eventual leakage of these molecules in after born life (by trauma, diseases, infections, inflammation, etc.), some of them having antigenic capability against the IS, may start a slow and subtle autoimmune-like response [21, 22] thus becoming one of the events contributing to the LGCI which strongly lies in the pathophysiology of DMPR [14, 15].

#### Morphological bases of the major components of the inner and outer blood retina barrier (iBRB and oBRB)

Human retina is isolated from the rest of eye cell populations by an elaborated continuous blood retina barriers which may be divided into inner, or vitreal side (iBRB) composed by Müller cell layer and the inner limiting membrane and the choroidal side or outer (oBRB), built by PEC, Bruch membrane and basal membranes and endothelia of *choriocapillaris.* The boundaries are sealed by the *ora serrata* in the periphery as described below.

##### Ultrastructure of Müller cells and its BM, the major components of the inner limiting membrane: 

**Fig. 1:**
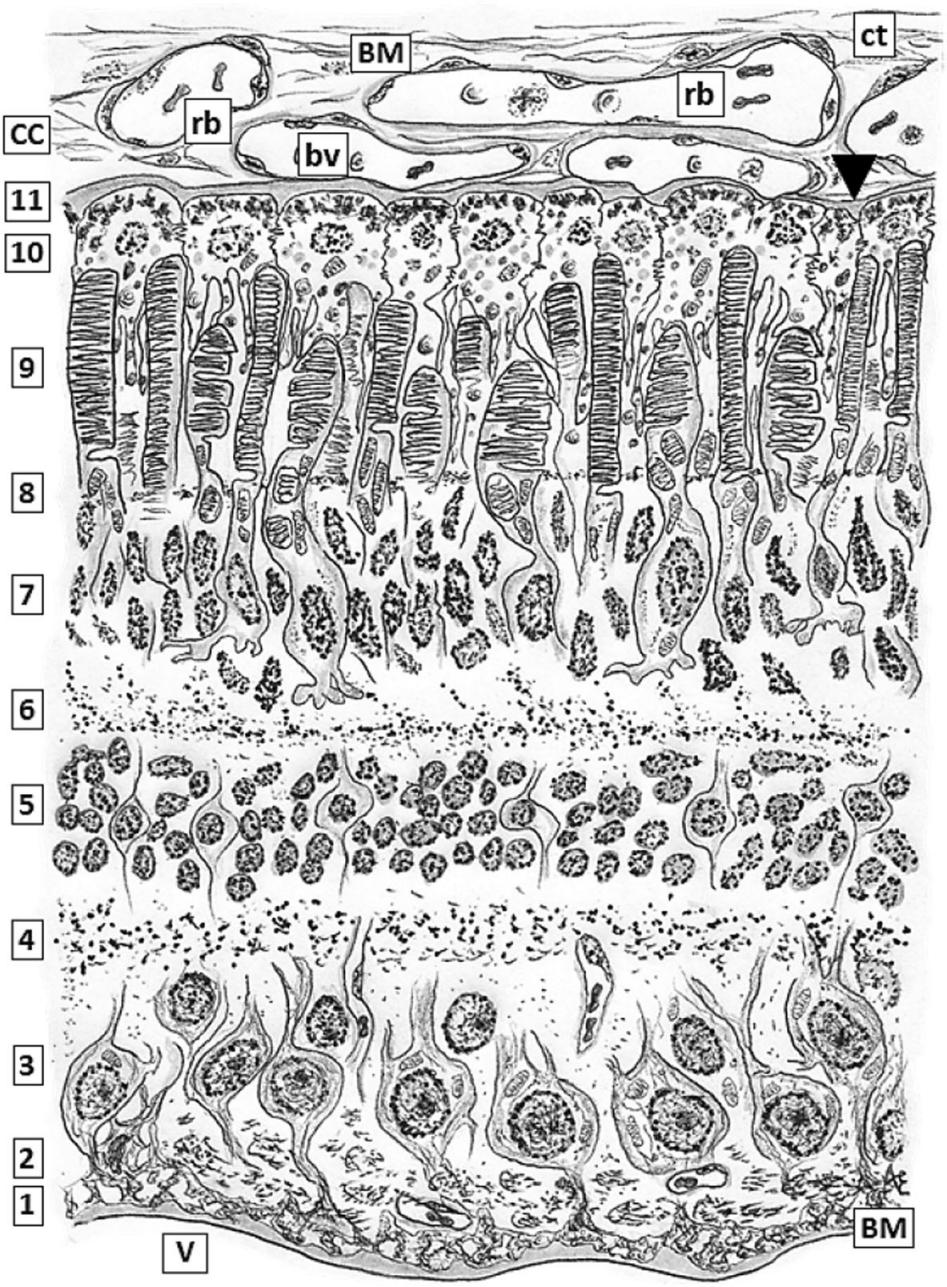
*Layers of the normal retina.* Low magnification: 1, inner limiting membrane bordering the vitreal body (V) constituted by a thin basal lamina, Bruch Membrane (BM). The opposite face of this BM shows delicate projections of Müller glial cells sealed each other by scattered tight junctions just above the BM; 2, layer mainly constituted by axons of ganglion neurons which form the optic nerve at the papilla;3, ganglion multipolar neurons layer; 4, inner plexiform layer; 5, inner nuclear layer; 6,outer plexiform layer; 7, outer nuclear layer; 8, outer limiting layer where abundant Zonulae adhaerentes (ZA), belt- shaped, are located between the photoreceptor neurons and the tinny terminals of Müller’s glial cells; 9, inner and outer segments of rods and cones layer; 10, pigment epithelial cells with varieties of junctional complexes between them . Richness in tight junctions plus normal integrity of Bruch membrane constitute the major morphological bases for the blood-retinal barrier; 11, Bruch membrane, a thin basal membrane (indicated with a black triangle, ▼) that adjoins to the extracellular spaces of chorio-capillaris (CC) or Choroidea, the medium layer of the eyeball showing abundant small blood vessels (bv) mainly fenestrated capillaries with a thin continuous BMs and venules containing scarce red blood cells (rb) distributed within scarce loose connective tissue (ct)

Among the *somae* and their intricated prolongations of different neuronal populations, there are intermixed the glial projections of Müller cells, whose dendriform projections play a very important role isolating neurons *somae* and their prolongations thus establishing the precise neuronal distribution in the different layers of the neural retina. Projections of Müller cells sealed by tight junctions (zonulae occludens, ZO) zonulae adherens (ZA) and gap junctions plus its own BM (inner limiting membrane) conform the morphological base of iBRB, sequestering retina cell populations from the vitreal environment (Fig. 1) [34–37].

##### Ultrastructure of pigment epithelium cells (PEC) and choriocapillaries, main components of the outer limiting membrane:

**Fig. 2:**
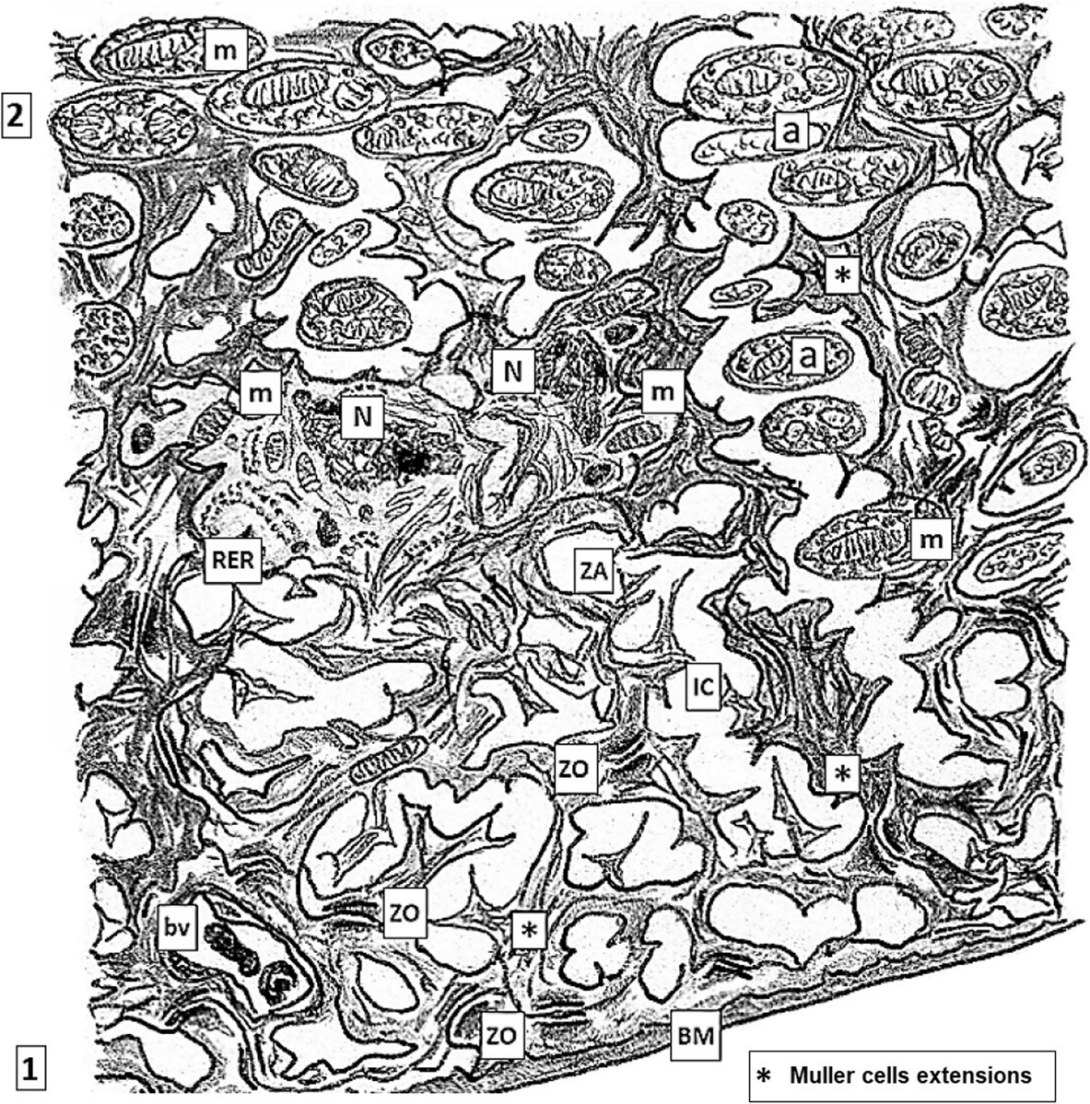
*Higher magnification of the layers 1 and 2 of normal retina as depicted in* Fig. 1*.* It is mainly constituted by axons obliquely sectioned (a) of ganglion neurons and inner limiting layer with nuclei (N) of two Müller cells and their delicate extensions (*) containing some organelles (mitochondria –m-, RER) and neurofibrils, leaning on the inner Bruch membrane (BM). Some few small blood vessel (bv) are identified. Developed tight junctions complexes (zonulae occludens and adherens, ZO, ZA) plus normal morpho-functioning of Bruch membrane are the main morphological bases for the vitreal-retinal barrier. Dilated intercellular spaces (IC), although drawed here as usual artifacts induced by processing, may become susceptible areas for the onset of microedema and then progression to cystoid degeneration (compare with Fig. 4)

**Fig. 3:**
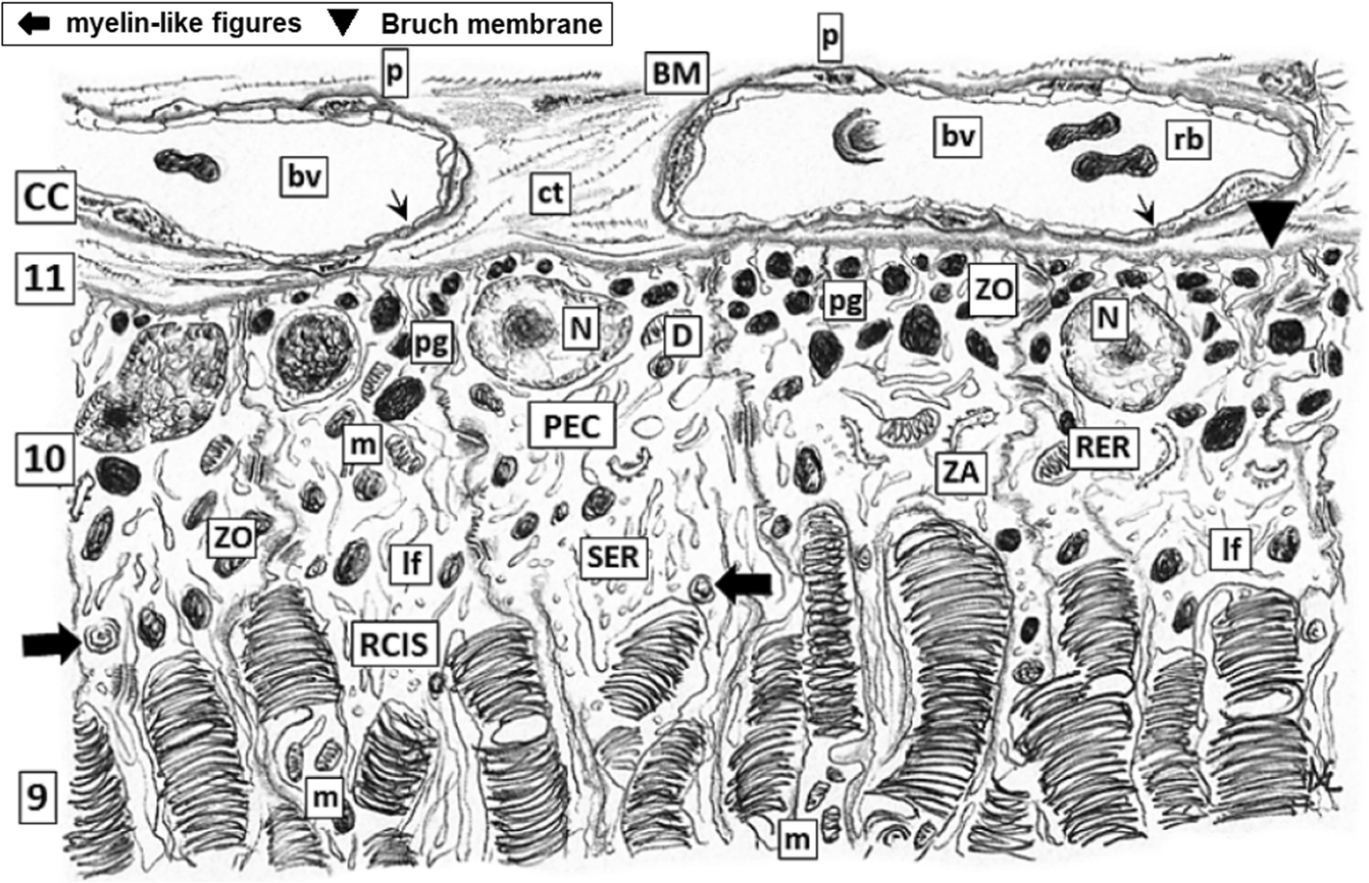
*Higher magnification of the layers 9 to 11 plus CC of normal retina as depicted in* Fig. 1*.* Fenestrated endothelia (arrows) with thinny BM and some pericytes (p) of bv within loose ct of CC layer which locate closer to Bruch membrane (indicated with a black triangle, ▼).; rods and cones inner segments (RCIS) are deeply allocated within entangled thin processes of PEC . The basal plasmalemma of PEC, underlying the BM, have complicated recesses sealed by ZO, ZA and desmosomes (D). Within these processes abundant pigment granules (pg) and lysofagosomes (lf) are visualized intermixed within a vast and intricated network of cisterns and vesicles of smooth endoplasmic reticulum (SER). Few aged sacs of rod and cones partially digested appears as myelin-like figures (filled black arrow)

A single layer of PEC lies on Bruch membrane being this its own basal membrane (BM). BM of PEC is in close contact with small blood vessels and capillaries running throughout the choroid *choriocapillaris* network whose endothelial cells are fully sealed by continuous ZO and ZA turning not permeable this boundary as also happens with those capillaries forming the basis of the whole hemato-CNS barrier [38, 39] (Fig. 2). It is worth to mentioning that, in contrast, there is not BM between PEC projections and photoreceptors (PRs) outer segments of rod and cones [40]. PEC are tall and very thin with numerous prolongations having a supranuclear Golgi. Both PEC and Bruch’s membrane are as slender as 15–20 μm thick. PEC is a very particular population since has highly specific appetite to continuously phagocyte aged, worn out or damaged membranous disc tips of the OS of PRs [41]. Prolongations of PEC build up a dense net of interdigitations among the OS of cone and rods. Cytoplasm of projections are filled with mitochondria indicating a high demand of energy for active molecules traffic. Contiguous surfaces of PEC digitations are continuously sealed by arrays of junctional complexes (ZO, ZA and gap junctions) [34, 35, 42]. These ultrastructural differentiations are strongly reminiscent to the assemble of junctional complexes observed in Sertoli cells surrounding maturing spermatogonia progenies, where reside the morphological bases of the hematotesticular barrier [43]. The apex of PECs prolongations totally stuff the interstitium among the slender OS of rods and cones building capsule-like cylindrical processes and intricated delicate prolongations. These PEC processes contain abundant melanin bodies and variable amounts of myelin-like (lamellar) bodies which are debris of phagocytosed aged OS rests of rod and cones in variables processes of digestion, having phospholipases activities [44–46] releasing LCHU-PUFAs to be reutilized in plasma membrane synthesis as well as pro-oxidant moieties [47]. PEC prolongations show a rich network of cisternae of agranular ER. Hence, the BM of PEC *plus* the complex array of ZO, ZA and tight junctions which seal the intercellular spaces conform one the major barrier of the retina on the choroidal side. In addition, *choriocapillaris* possesses continuous endothelial cells and BM, without fenestrations being per se highly impermeable. Prolongations of PEC and BM of *choriocapillaris* are in closed and uninterrupted contact thus fully sealing retina with respect the choroidal side (Fig. 3) [34, 35, 42, 48, 49]. So, leakage in and out from choroid blood vessels and retina is normally avoided. This is important since many retinal antigenic determinants expressed in later prenatal period and many others molecules to be eventually expressed along the life span of the subject [22, 33, 41, 50] will remain sequestrated within retina environment without further contact with the IS of the individual in normal conditions.

#### Disruption of BRBs in DMRP

Several research shows that PEC becomes altered in DM allowing increased leakage from choroid blood vessels together with delayed reabsorption of extracellular fluids being this one of the causes of retinal edema [51]. It is generally assumed that the disruption of the iBRB is a major cause of DMRP [36, 52] but there is also evidences that the disruption of the external limiting membrane and PEC causing oBRB damage also contributes to the pathogenesis of DMRP as observed in diabetic models [53–55]. In an experimental model of type 2 DM, the GK rats, oBRB damage is linked to the formation of clefts through the PEC prolongations which enables diapedesis of inflammatory cell between the retina boundary and choroid [50]. In addition Müller glial cells show increased ballooning [56] due to ER and mitochondrial swelling which are constant ultrastructural findings in DM [57] and also when ω3 and ω6 PUFA deficiency (w3/w6D) occurs [58]. Actually, slow dissolution of inter-vascular junctions, which result in vascular leakage and retinal edema favors LGCI [59].

##### w3/w6D induces disruption of several cellular barriers increasing the leakage of macromolecules from blood vessels into extravascular environment:

Consistent research in animals and humans suffering of variables degrees of nutritional or metabolically w3/w6D result in a wide cellular and molecular abnormalities of key molecules involved in several cell–cell, cell-BM adhesion and consequently breakdown of several limiting barriers also linked to the appearance of abnormal fatty acids (FA) as previously reported [58, 60–63].

Once metabolically, or nutritional, w3/w6D is established, a progressive breakdown and leakage appears in many epithelial/stromata barriers, which in turn become propitious scenarios to the slow developing of LGCI [64]. Two examples will be briefly described since some morpho-functional similarities exist regarding BRB among retina tissues and several epidermoid and urothelial epithelia interphases. In rodents increased loss of water through skin, with increased hyperplasia, hyper- and para-keratosis and scaliness is observed in epidermis and other Malpighian epithelia as esophagus and forestomach, indicating damage to the water barrier function [65]. Reduction of the number of desmosomes has also been reported in the small bowel intestinal epithelia, forestomach and esophagus [46, 65, 66] resulting in abnormal gastrointestinal absorption in w3/w6D [67]. Blood vessels in dermis and in other organs of w3/w6D animals showed vasodilatation with notorious extravasation of lymphocytes, monocytes and polymorpho nuclear leukocytes (PMN) [58, 60, 68, 69]. In these barriers paracellular permeability is mainly regulated by the structure and functions of occludins tight junctions and ZO and ZA. Occludins interacts each other in contiguous cells building the major barrier in several endothelia being a main key array for the blood–brain barrier and for BRB in the eye, too. Desmosomes represent one of the key cell–cell adhesion mechanisms in epithelia, endothelia, cardiomyocytes, and particularly in Malpighian epithelia (as skin, esophagus, corneal and *ora serrata* in the eye) among others [70]. Desmosomes are composed of desmoglein 1, 2 and 3 together with desmocollins 1, 2 and 3, collectively named as desmosomal cadherins [71]. When E-cadherin negative epithelial cells are cultured with ω6-GLA, a LCHU-PUFA, tighter cell-cell association developed when compared with controls. Immunocytochemical and electron microscopic studies revealed that this adhesion was mediated by desmosomes showing heavy marking for desmoglein [72]. On the other hand, ω3 and ω6 PUFAs regulate cell–matrix adhesion, an important crossroads for morpho- and functional integrity of blood-tissues barriers as a whole [62, 73, 74].

**Fig. 4:**
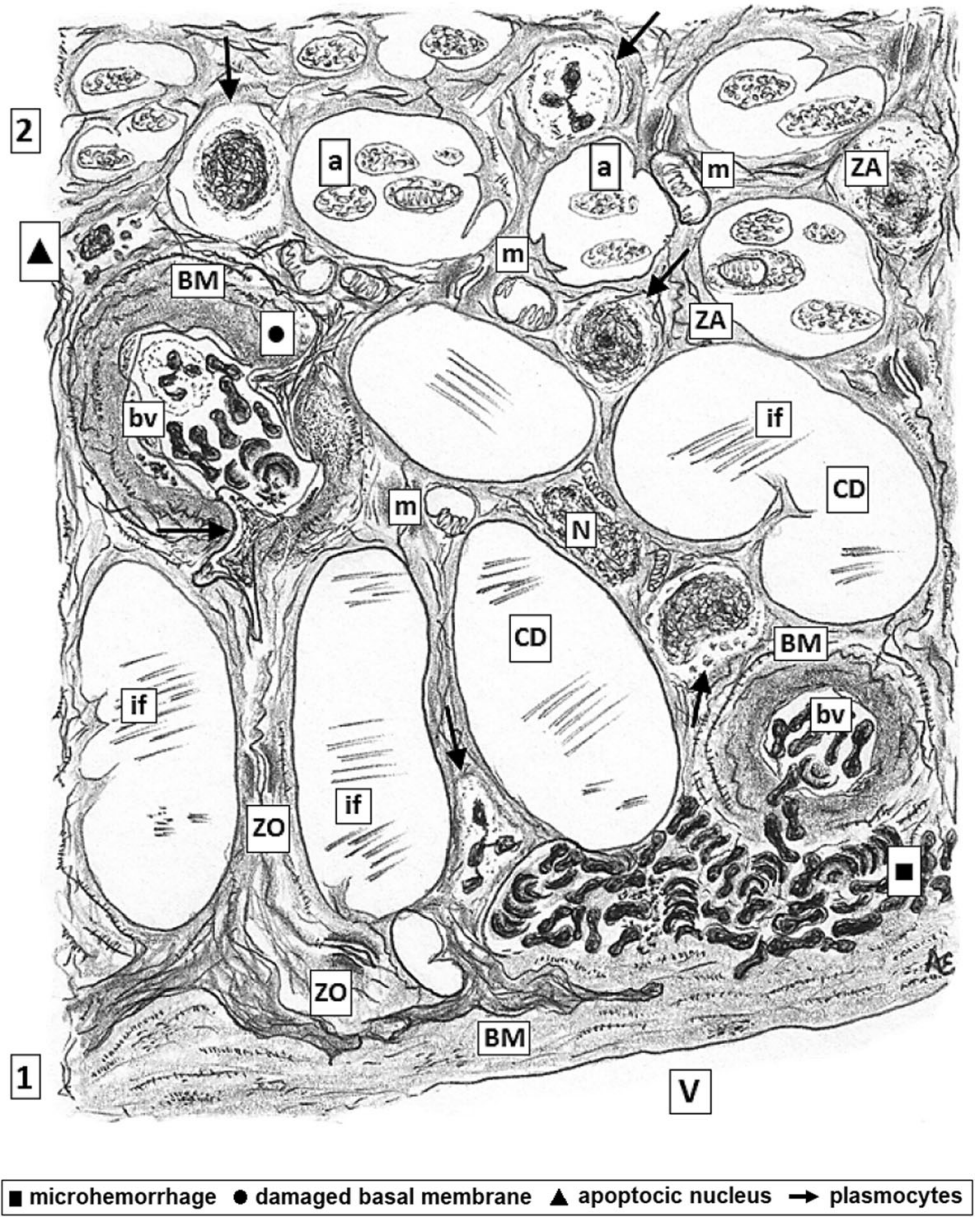
*Human retina of diabetic patient with advanced DMRP.* This picture illustrating layers 1 and 2 as identified in Fig. 1. Also compare with Fig. 2. Microhemorrhage (indicated with a black square, ■) from heavily congested bv pouring out rb and inflammatory cells within augmented perivascular collageno-genesis in ct interstitium. Cystoid degeneration (CD) appears as enlarged and coalescent bubbles arising within IC containing coarse bundles of intermediate filaments (if). Thickened Bruch membrane bordering the vitreous body and BMs of bv are characteristic features of DMRP diapedesis of inflammatory cells (polynuclears, lymphocytes) and wandering plasmocytes are pointed (arrows). Swollen mitochondria are frequently seen in axolemma of the layer 2 of axons of ganglion neurons and cytoplasmic projections of Müller glial cells overloaded with coarse bundles o neurofilaments some of them invading the lumen of bv through damaged BMs (indicated with a black circle, ●) whereas others are deeply anchored within Bruch membrane. Incontinent or damaged ZO and ZA are shown. Apoptotic Müller cell nucleus (indicated with a black triangle, ▲)

**Fig. 5:**
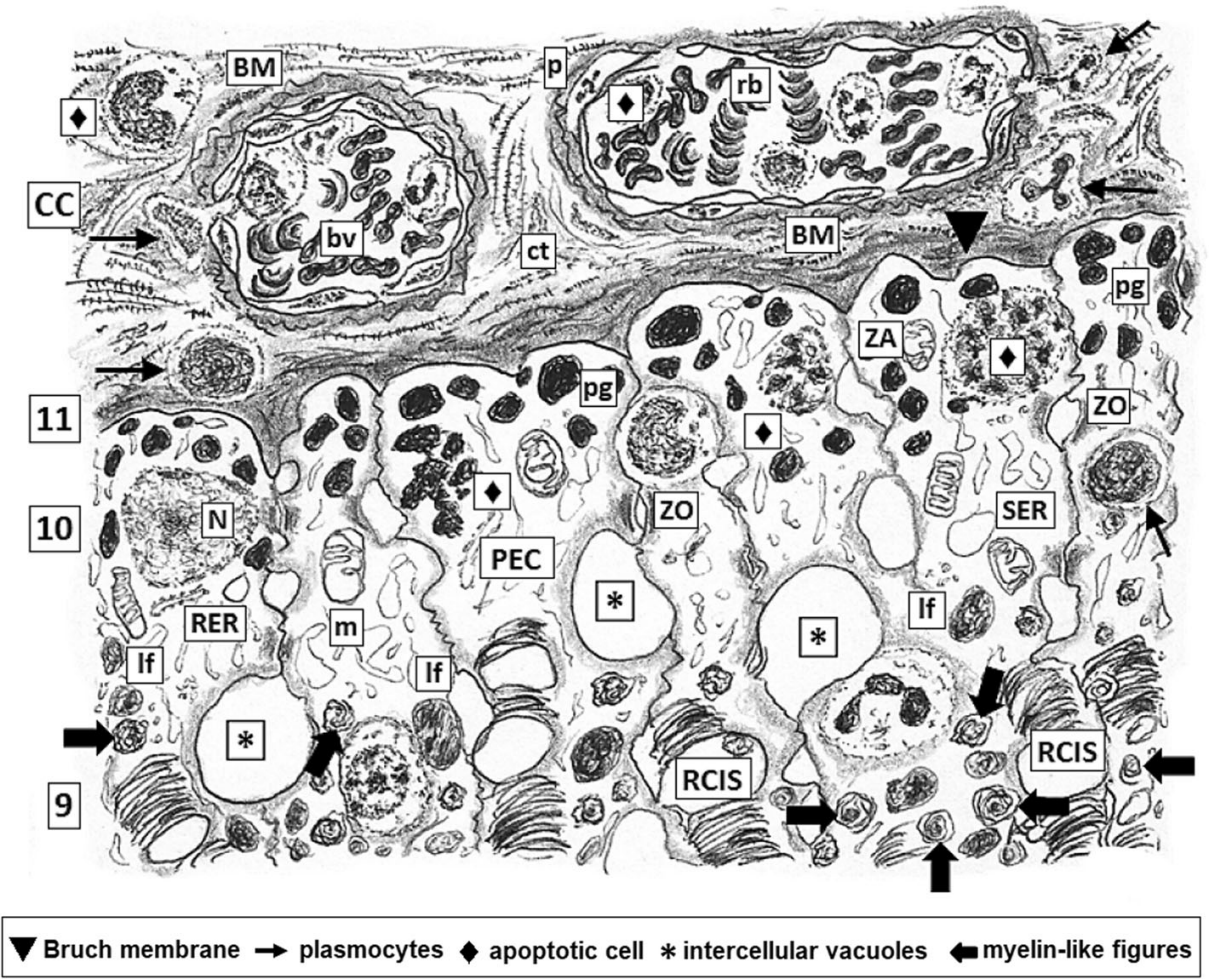
*Higher magnification of the layers 9 to 11 plus CC of human retina of diabetic patient with advanced DMRP; as identified in* Fig. 1*.* Also compare with Fig. 3. Congested bv in CC layer showing increased perivascular collagen genesis in coarse ct interstitium. Thickening of Bruch basal membrane (indicated with a black triangle,▼) bordering against CC layer and BMs of bv are characteristic features of DMRP at this stage. Diapedesis of inflammatory cells (polynuclears, lymphocytes) from bv and wandering plasmocytes are pointed (arrow). There are scarce pericytes some of them in apoptosis (apoptotic cells were indicated with a black diamond, ♦). Swollen mitochondria and vesicles of SER are frequently seen in cytoplasm of PEC, whose nuclei are in diverse stages of apoptosis. Incontinent or damaged ZO, ZA are identified thus facilitating the development of large PEC intercellular vacuoles (*) which in turns collaborate in the worsening of retinal edema. Few rods and cones inner segments (RCIS) are visible, most of them degenerated and vacuolated. Closer PEC recesses are filled with increased number of lyso-phagosomes (lf) and myelin like figures (indicated with a filled black arrow) containing aged sacs membranes in diverse stages of digestion

When inflammation process starts the first steps is vasodilatation with slowing down of the erythrocytes flux and adhesion to the surface of endothelium being now ZO and ZA among endothelial cells the major barriers in charge of control the input and output of several molecules (Figs. 4 and 5). Members of the ω3 PUFAs family are able to modify certain functions of the tight junction in vascular endothelial cells, as the trans-endothelial resistance and the paracellular permeability [75, 76].

The other example of barrier disruption and the role exerted by LCHU-PUFAs, is located in the luminal surface of mammals urinary tract built by urothelial umbrella cells which totally covers pelvises, ureters and bladder and are strongly modified by PUFAs composition [64]. Luminal urinary surface showed polygonal areas of big clustered glycoproteic particles, the uroplakins, which show a strong morphological resemblance to visual pigments, heavily packed in PM of retinal rod and cones. Along with ZO, ZA and other cell-cell union complexes, the PM built the main morphological component of the “permeability barrier” of urothelium [77, 78]. So, leakage of putative antigens, mutagens, and other toxic molecules from urine into the chorionic blood vessels is halted. However, umbrella cells require of a fast turnover of PM, as also happens in retinal rod and cones, being these continuously synthesized with a constant high requirement of essential PUFAs. The ω3 and ω6 LCHU-PUFAs play a key role in the maintenance of the molecular structure and functions of urothelium barrier against putative pathogenic and injurious molecules carried by urine [79, 80].

Cited examples of intake/administration/metabolism disbalances of LC-PUFAs strongly evokes similarities with scenarios on eye BRBs, the turnover of the highly specialized plasma membrane of OS PLs of rod and cones and their high demand of ω3 and ω6 LCHU-PUFAs.

#### Ultrastructure of rod and cones and roles of membrane PUFAs for photoreceptors

Rods are tall, long and thin highly specialized neurons ordered in palisade array whose apical side are choroid-oriented to the apex face of PEC (Figs. 1 and 3). Rod axons establish synapses with other neurons in the innermost layers of the retina. Their apical segment, as happens in cones too, is highly differentiated in two segments: outer (OS) and inner joined by a thin portion of cytoplasm. The OS of rods contains the integral membrane glycoprotein (GP) rhodopsin. OS is heavily loaded with a large number of closed membranous sacs, which overlap as pancakes stacked in a perpendicular array respect to the major axis of the rod. Rhodopsin and others GPs visual pigments move along and within (flip-flop) the phospholipid bilayer thus being their functionality fully dependent of the viscosity/fluidity balance of the sacs membranes, fact which in turns is mainly determined for the relative ratio of ω3/ω6 /ω9 PUFAs and cholesterol [81, 82]. Rods are distributed by the retina together with the cones but are better adapted for vision with little light intensity or twilight vision (scotopic vision), which does not allow a proper the perception of colors. Interestingly, this kind of crepuscular vision remains better preserved in DMRP [83, 84].

Cones make up a population of about 120 million of thin, highly polarized neurons, about 100 μm in length, with their major axis perpendicular to the surface of the retina and also encastrated within complex interdigitations of PEC. Cones gathers in the *fovea centralis,* an area without blood vessels where the remnant layers of the retina are very thin being towards this zone where our eyes clearly project the objects of the outside world (photopic vision). Worth to consider, incoming and outcoming bunch of eye blood vessels and nerves are morphologically isolated from retina cell population by elaborated complex union as previously described built by oligodendroglia, pericytes and impermeable blood vessels of the CNS. Proinflammatory bioactive lipids (BLs) derivative molecules, as eicosanoids, leukotrienes (LTs) and cytokines released from macrophages, endothelial cells and other cells, may migrate from small blood vessels and choriocapillaris crawling towards the choroids and retina when the selective barriers described above became progressively damaged. These proinflammatory molecules have facilitated their access to the neighbors OS of cone and rods due to the lack of a distinctive basal membrane between retina and choroids.

### Metabolism of PUFAs and DMRP

#### PUFAs in health. PUFAs metabolism is altered in DM

**Fig. 6:**
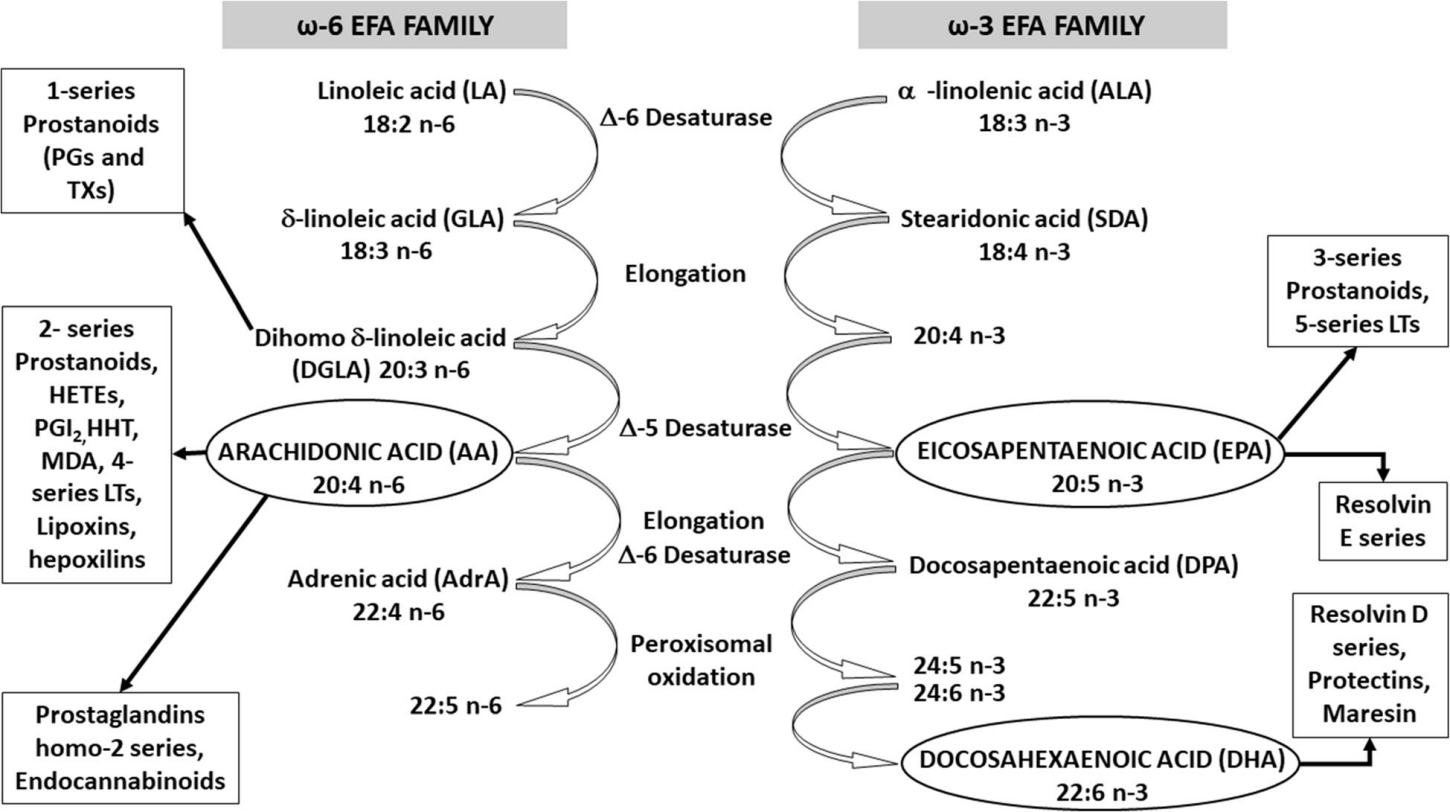
*Metabolism of Dietary Fatty Acids*. FA are oxidized to provide energy, stored in adipose tissue, and selectively incorporated into phospholipids (PL) of all cellular membranes. Once ingested in food, they are desaturated and elongated to yield several PUFAs. PUFAs are long carbons- chained molecules having two or more double bonds of the cis configuration. ω3 and ω6 PUFAs cannot be synthesized by metazoan. They must be ingested through the diet and hence are named Essential Fatty Acids (EFAs). PUFAs ω6 derive from LA (18:2 ω6) and ω3 PUFAs arise from alpha- linolenic acid (ALA, 18:3 ω3). On the contrary, monounsaturated palmitoleic acid (POA, 16:1 ω7) and oleic acid (OA, 18:1 ω9) are easily synthesized by the body. Even though all EFAs are PUFAs not all PUFAs are essentials. In this work PUFAs and EFAs will be used as synonymous. Non-EFAs refers to monounsaturated POA and OA and their non –EFAs long chained PUFAs derivatives; also saturated fat, trans FA and cholesterol are included under this name. ALA and LA- and in certain conditions, non-EFA from the ω7 and ω9 families- compete for a common ∆5- and ∆6-desaturase and cyclo-oxygenase and lipo-oxygenase enzymes kits that are essential for the formation of long-chain metabolites such as AA, EPA and DHA from the EFAs linoleic and alpha-linolenic acids, respectively. Resulting bioactive lipids (BLs) have key, oftenly opposite, biological functions [28]. In this “race”, 18:3 ω3 ALA is desaturated preferentially, followed by 18:2 ω6 LA, thus avoiding the conversion of OA to the more highly unsaturated ω9 metabolites

As depicted in Fig. 6 and briefly explained in the legend, PUFAs have at least two major functions: as a major component of cell membranes bilayers and as precursors of many BLs. Under normal physiological conditions most of BLs derived from ω6-AA, ω3-EPA and ω3-DHA as lipoxins, resolvins, and protectins tend to maintain normal homeostasis and avoid the initiation of LGCI in DM [85, 86].

Relative metabolically/functionally w3/w6D may occur more often than believed. This deficiency may occur if one or more of three circumstances appear: 1- lack of essentials ω3/ω6 PUFAs intake in the diet, likely to be seen in less developed societies; 2- unhealthy intake of fats and lipids in the diet, such as excess consumption of saturated FA, hydrogenated vegetable oils and other non-Essential Fatty Acids (EFAs), mainly ω9 precursor (i.e. transgenic corn/sunflower oils enriched in oleic acid,OA, 18:1 ω9) which vol/vol compete with ω3/ω6 PUFAs metabolism (see legend Fig. 6). 3- Abnormalities in the metabolism of FA, as certainly happens in DM, as will be described. Although industrial societies have almost eradicated the w3/w6D from insufficient intake of EFAs, items 2 and 3 become risky situations for both rich and poor countries, with the aftermaths of metabolic syndrome, obesity, dyslipidemias, DM and their late complications as DMRP [1, 2, 4, 87].

**Fig. 7:**
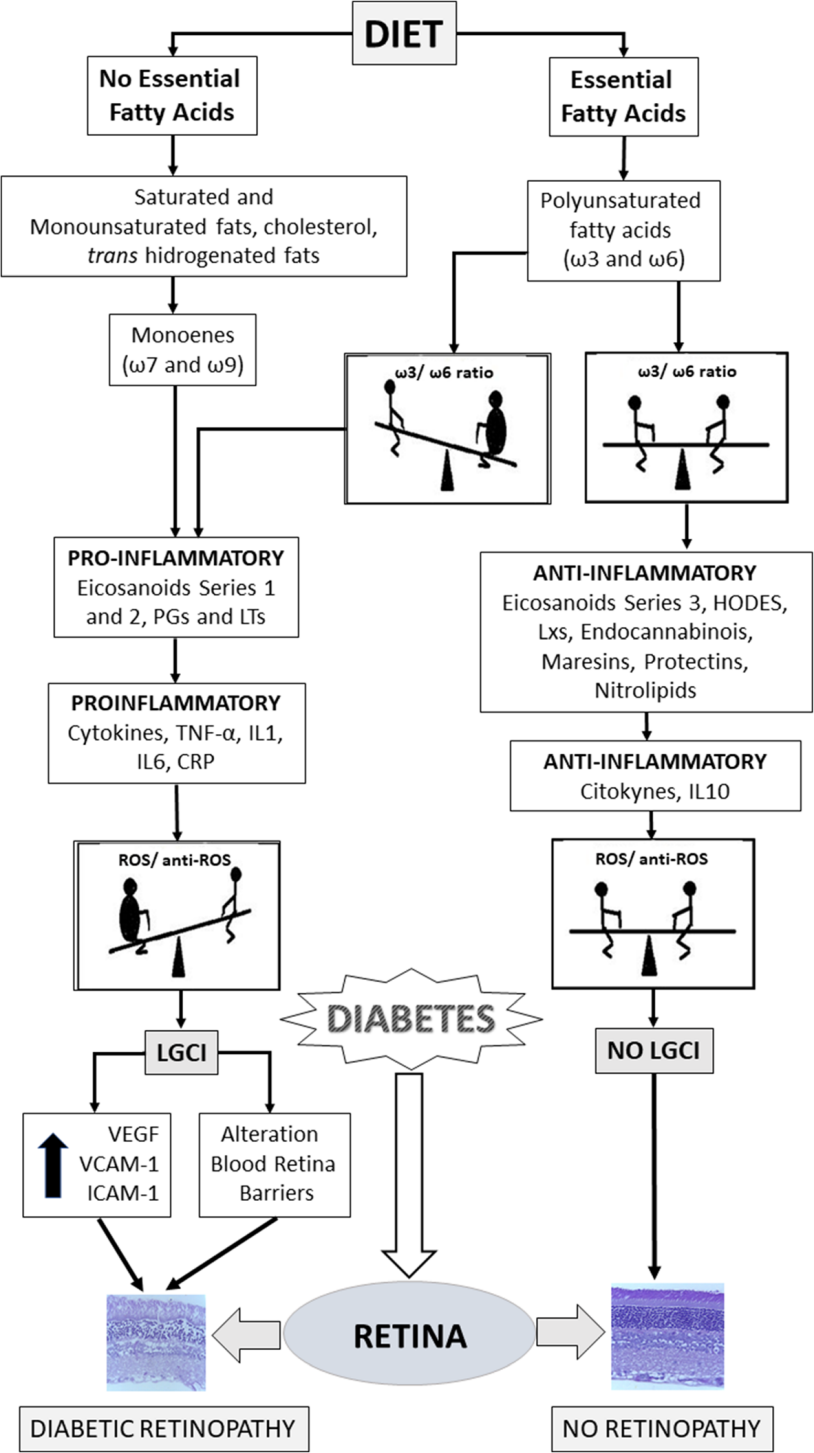
*Fatty acids, inflammation and diabetic retinopathy.* Role of dietary fatty acids, bioactive lipidic metabolites and LGCI and to risk for diabetic retinopathy

We showed that abnormal LCHU-PUFA, like eicosatrienoic acid (20:3 ω9) produced by an imbalance in metabolism of different series of FA, a marker of w3/w6D, was able to down-regulate the expression of both E-cadherin and desmoglein in squamous skin cells, key molecules for the adequate sealing of epithelial-conjunctive barriers [61, 88]. As discussed, when a metabolically w3/w6D occurs there is weakened expression of cell adhesion molecules and cell–cell adhesiveness, which appears to be involved in certain pathological features as losses of functions of several barriers involving endothelia, cell- cell and cell-matrix interactions as described above (summary in Fig. 7).

LCHU-PUFAs together with cholesterol can regulate membranes particular properties as fluidity/viscosity and in turns they modulate their dynamics and biophysical properties [89] and lateral segregation of membrane glycoprotein as happen with visual pigment of rods and cones. The visual macromolecules of the pigment must be densely packed, ensuring an optimal exposure to the photons, for their correct functioning so changes in the membranes can affect this arrangement [90–92].

In DM the metabolism of PUFAs is clearly abnormal, insofar as there is a partial loss of the enzyme delta-6-desaturase activity, which catalyzes the initial desaturation step in the pathways involved in the synthesis of longer chain PUFAs, whose disponibility becomes progressively diminished [15, 28, 93, 94]. Gong et al. (2017) reviewed these issues concluding that dietary ω3-LCHU-PUFAs reduce retinal and choroidal neo-angiogenesis. ω3-LCHU-PUFAs BLs metabolites from COX2 and LOX are generally inhibitors whereas ω6-LCHU-PUFAs metabolites promote inflammation and angiogenesis. However, the ω3 and the ω6 lipid products of cytochrome P450 oxidase 2C promote neovascularization in retina and choroid, suggesting that inhibition of this pathway might be beneficial in prevention and treatment of DMRP [8].

#### PUFAs alteration in DM resembles w3/w6D

As quoted above, a relative deficiency of ω3 EPA and DHA and ω6 AA, or excesses of ω9 food intake, may predispose to the development of DM. Patients with types DM1 and DM2 have decreased plasma and tissues concentrations of AA, EPA and DHA whereas ω9 FA derivatives are increased in their plasma phospholipid fraction [1, 4, 95]. These findings give further support to the concept that disbalanced metabolism of PUFAs may have a significant role in the pathobiology of DM and their complications in membranes phospholipids heavily dependent of ω3 and ω6 LCHU-PUFAs supply, as is mammal retina [14, 18, 96].

#### PUFAs changes in PR membranes in DM and ω3 and ω6 deficiency (w3/w6D)

A strong association between dyslipidemia and the development of diabetic retinopathy was revealed by results of the Diabetes Control and Complications Trial/Epidemiology of Diabetes Interventions and Complications cohort study [97].

In vivo experiments in Rhesus monkeys shows that a diet low in ω3 PUFA (ALA or DHA) was associated with specific perturbations in retinal function, including increased implicit times and a substantial delay in the recovery of the rod-isolated photoresponse. Rod sensitivity was reduced by 40% in the long standing dietary ω3–deficient monkeys and the onset of the rising phase of the photoresponse and rod recovery were also significantly delayed [98]. These alterations appear to be linked to dimminution in retinal DHA levels that may alter biophysical properties and lipid-protein interactions in retinal membranes where visual pigments are inserted.

Early-stage diabetes induced a marked decrease in elongases expression. These fact produce a significant reduction in total retinal DHA and diminished incorporation of LCHU-PUFAs into retinal phosphatidylcholine. The decrease in ω3/ω6 PUFAs ratio in retina is associated with an increase in gene expression of proinflammatory markers IL6, VEGF, and intercellular adhesion molecule-1 (ICAM-1) thus creating an LGCI environment that contributes and maintains the development of DMRP [14, 18].

In addition, deficiency of LCHU-PUFAs, and low ω3/ω6 PUFA ratios occur in human retinas with macular degenerations. Quality of lipids foods certainly influence human retinal ω3/ω6 ratios, which may explain why a diet high in ω3 rich fish oil (DHA and EPA) is beneficial against macular degeneration as pointed out in some epidemiologic studies [81, 99].

Streptozotocin-diabetic rats showed decreased desaturase and elongase activities and alterations in rod function exacerbated by low intake of dietary ω3 PUFAs. A decrease in retinal DHA was found (171%) in diabetics animals fed deficient ω3 diet [8, 96].

In summary, some epidemiology, clinical and experimental research coincidently show that a decrease of retinal ω3 levels, increase of oxidative stress and proinflammatory metabolites are common factors that are involved in the pathophysiology of photoreceptor death in several degenerative diseases of the retina including DMRP. On the contrary high levels of DHA synthetized by retinal metabolism protects retina PRs from apoptosis induced by oxidative stress and promotes their differentiation [8, 17].

#### PUFAs in rod and cones outer segments. Perturbations in DM

In normal conditions, LCHU-PUFAs are particularly enriched in vascular retina, being the bulk of DHA and AA around 10% of isolated membranes of retinal capillaries and surrounding pericytes [100]. Radiolabeled ω3 LCHU-PUFA precursor given intraperitoneally or orally to healthy rats is first localized in the liver and one hour later labeled DHA was detected in rods. DHA and docosapentaenoic acid (DPA, 22:5 ω6) are avidly incorporated in PRs membranes of rods and cones reaching a peak at 24 h after infusion. PRs incorporate ω3 and also ω6 LCHU-PUFAs 3–5 times more efficiently that PEC does [101, 102]. LCHU-PUFAs are then convoyed to the smooth endoplasmic reticulum (ER) located in the myoid (the basal area of the OS) of PRs and then into a variety of membranes PLs and triglycerides. LCHU-PUFAs enriched PLs are used for synthesis of new membranes of disk and vesicles of OS of rods and cones and tenaciously retained in the bilayer closer to the molecules of rhodopsin and to the other visual pigments. Damaged or aged disks slowly migrate contacting the intricated cytoplasmic extensions of PEC where they are engulfed and digested within PEC lysosomes (see Figs. 1 and 3). DHA and other ω3 and ω6 LCHU-PUFAs are separated and esterified in triglycerides, stored in the abundant oil droplets of PEC and then re-uptaked by myoid area of rod and cones [8, 12, 82, 103, 104].

These highly and selective needs for ω3 and ω6 LCHU-PUFAs for efficient morpho-physiology of retina highlights the sensible dependence of enough supply of dietary precursor (the EFAs ω3-ALA and ω6-AA) of these LCHU-PUFAs. Even though the human body (mainly the liver) potentially have the capabilities to desaturate and elongate EFAs for satisfice the needs of highly differentiated retinal populations, these are unusually high (see Fig. 1). Accordingly, when an experimental deprivation of ω3 and ω6 LCHU-PUFAs is induced these lipids are strongly retained by retinal PRs; if this deficiency is maintained finally the visual acuity become altered [105, 106]. Unfortunately, the food sources of formed LCHU-PUFAs, particularly those enriched in ω3 LCHU-PUFAs, are very scarce in the so called western diets thus supporting the need of dietary intervention or nutrient supplementation, even in healthy pregnancy, childhood and elderly [67, 107]. Hence, if the desaturation and elongation of ω3-ALA and ω6-AA become abnormal, as happens in several metabolic diseases including DM, the fresh supply of ω3 and ω6 LCHU-PUFAs for rod and cones become progressively deficitary. Progressive blockade of activities of Δ9, Δ6 and Δ5 desaturases have been consistently described in DM animals and humans as said before [108, 109]. Actually, DM patients shows lower concentrations of LCHU-PUFAs whereas saturated FA and monoenes, mainly OA and derivatives, are higher [110]. In a DM rat model, a decrease in FA elongases activities were observed with decreased incorporation of LCHU-PUFAs in retinal PLs and increased markers of inflammation [14, 18]. Promisingly DM2 middle-aged and elderly patients enroled in the “Prevención con Dieta Mediterranea” (PREDIMED) study, after 6 years of follow up those adherents to Mediterranean dietary habits, particularly those whose foods included ω3-LCHU-PUFAs (around 500 mg/day), showed prolonged preservation of retinal neuronal functions [111]. For practical purposes to the clinician a well summarized table of the putative beneficial actions of ω3-LCHU-PUFAs supplementation in DMRP and its major complication, the age related macular degeneration, is giving by San Giovanni and Chew (2005) [12].

#### Autoimmune response and disruption of blood retinal barrier (BRB) in DM

Since each of the three embryonic layers of the eye will differentiate in particularly cell populations, a vast number of new epitopes will appear throughout the pre- and post- natal development, some of them being highly sensible if recognized by the IS of the own subject, thus becoming propense to trigger an autoimmune disease in the eye globe. When inner and/or outer BRB become disrupted, these “not-recognized” molecules become target sites for the IS response. BLs as eicosanoids, may differentially affect the activities of such molecules, as ILs, distorting their functions [12, 112, 113]. Coincidently, lower levels of anti-inflammatory IL-10 were comparatively detected in plasma and tissue B lymphocytes of patients suffering of DMRP [114]. In a model of DM rats BRB permeability, assayed with Evans Blue (EB) dye, it was altered at six months of disease, whereas the amount of tight junction proteins decreased significantly. The treatment with TNFSF15, an endogenous neovascularization inhibitor and strong negative regulator of vascular homeostasis, protected the BRB functions [115]. In advanced stages of DM, measures of TL1A levels, a variety of proinflammatory TNF-α, in the retina and vitreous were significantly increased. In addition, values for VEGF, TNF-α and IL-1β in the retina and vitreous were comparatively higher at 3 and 6 months in the DM [23]. As illustrated (Figs. 3 and 5) pericyte also contributes to impermeability of the BRB. Actually, it was showed that pericyte apoptosis in diabetes may cause a subtle abnormal immune response as demonstrated by in vitro study pointing out that several retinal autoantibodies may induce pericyte death followed by the complement system activation [116].

#### Intraretinal low grade chronic inflammation (LGCI) in DM

Consistently experimental, cohort and clinical studies showed that in DMRP characteristics signs of LGCI develops. This issue has been thoroughly revised by Das [15, 28] and other authors [52, 117]. Indeed, under ophthalmoscopic examination increased diameter of retinal vessels with augmented blood flow and eventually blood stasis, followed by leakage of plasma are observed. These lesions are accompanied by proteins leakage, BLs release, leucocyte lodging, diapedesis and migration of lymphocytes. Microscopical examination of the retina allows to corroborate inflammatory cells colonization (Figs. 4 and 5). Attachment and diapedesis of inflammatory cells may be linked to increased synthesis of TNF-α, VEGFs, series-2 PGs, enhanced expression of ICAM-1 on the blood vessels bed. Also, expression of integrins, as laminin, fibronectin and vascular cell adhesion molecule-1 (VCAM-1) arise on leucocytes cell membranes. Taken as a whole this scenario also indicate augmented oxidative stress [118]. Rheological disturbances in the blood flow causes increased synthesis of several reactive oxygen species (ROS) and lipid peroxidation [119]. Eventually a slow and progressive loss of permeability of the BRB develops along with endothelial disfunction, followed by pericytes apoptosis and endothelial denudation. In a following stage retinal capillaries will suffer periods of ischemia followed by reperfusion [120]. Interesting, patients with rheumatoid arthritis also suffering of DMRP treated with anti-inflammatory NSAIDs as aspirin at higher doses showed comparatively less severe retinal histopathological alterations [121]. These effects may be due to diminution in the synthesis of COX-2 derivatives as PGs, prostacyclines, TXs and inflammatory COX2-endocannabinoids derivates [2, 32, 118, 120]. In addition DMRP patients exhibited reduced plasma and vitreal concentrations of two key vasodilators, PGE1 and PGI2 whereas TXB2 and LTs were increased along with high vitreous expression of TNF-α, IL1 and IL6 [122, 123]. As happens in other vascular beds in diabetic patients, in DMRP there is also a loss of homeostatic balance between vasodilator and platelet anti-aggregator effects against platelet aggregator and vasoconstrictors response induced by TXA2 and LTs, being this one of the causes of the alterations of ischemic vasoconstriction and reperfusion of retinal vessels, scenario aggravated by activation of platelet aggregation and clotting trend [10].

## Conclusions and recommendation for healthy practices in long standing diabetic patients in risk of diabetic- linked retinopathy

As analyzed, DMRP and its accompanying lesions constitute a frequent ocular complication in long-standing diabetic patients. These illnesses, along with other associated chronic diseases such as obesity, overweight, some cancers, several heart diseases, dyslipidemias and hypertension, have in common a persistent condition of LGCI [4, 15, 28, 93]. Once this scenario is generated, which usually takes a long asymptomatic time, an imbalance is established and maintained between a decreased synthesis of anti-inflammatory components such as some cytokines and bioactive lipids (as resolvins, maresins and protectins) derivatives of ω3 or ω6 LC-PUFAs, on the one hand, and simultaneously, a sustained increase in plasma (and intraocular) proinflammatory cytokines, PGs, pro-inflammatory eicosanoids, leukotrienes, certain growth factors (as VEGF), free radicals, ROS and several auto-antibodies. Abnormal or deficient availability of essential ω3 PUFA in foods and/or cell membranes, indeed take place due to unhealthy dietary practices as are consumption of foods very rich in saturated fats, or genetically modified sunflower oil enriched in non- essential ω9 oleic acid, and simple sugars, since a chronic subclinical or border-line w3/w6D may occur. Hence It seems beneficial to start with easy attempts devoted to stimulate those anti-inflammatory molecules that the organism naturally produces, finished apparently simple measures, sustained throughout the life span of the DM patients, in order to prevent and to attenuate the visual complications of long-standing DM (Fig. 7). Beside the scheduled controls with the ophthalmologist, these measures include, in the broad sense, a global lifestyle change, including a strict control of blood glucose avoiding sudden, extreme and continuous fluctuations in glycemic values, strict weight control, healthy dietary habits with low intake of fatty meats, saturated and/or processed fats, increased intake of vegetables and legumes and few lean meats. More meals containing “blue fishes” rich in ω3 (mackerel, salmon, herring, tuna, sardine, containing up 10% of fats), daily exercise of moderate intensity, such as cycling in the plain, or fixed bicycle, walks outside at a brisk pace, or on mechanical treadmills (5 km/h) for 30–45 min. Because the intake of ω3 rich-fishes is usually scarce in the West diet, due to cultural reasons, availability and / or higher costs, it is advisable exogenous daily per os administration of well- sealed fish oil capsules enriched in ω3-EPA and ω3-DHA, usually containing of 1000 mg, (recommended dose: 2000 mg/day), immediately before the meals.

## Abbreviations

AA: ω6-arachidonic acid
BLs: Bioactive lipids derivatives
BM: Basal membrane
BRB: Blood retinal barrier
CNS: Central nervous system
DH: ω3-docosahexaenoic acid
DM: Diabetes mellitus
DMRP: Diabetes mellitus- linked retinopathy
EFAs: Essential fatty acids
EPA: ω3-eicosapentaenoic acid
FA: Fatty acids
IL: Interleukin
IS: Immunological system
LCHU-PUFAs: Long chained highly unsaturated polyunsaturated fatty acids
LGCI: Low grade chronic inflammation
LTs: Leukotrienes
OBRB: Outer blood retinal barrier
OS: Outer segment
PEC: Pigmented epithelial cells
PGs: Prostaglandins
PLs: Phospholipid
PM: Plasma membrane
PRs: Photoreceptors
PUFAs: Polyunsaturated fatty acids
VEGF: Vascular endothelial growth factor
w3/w6D: ω3 and ω6 PUFA deficiency
ZA: Zonulae adherens
ZO: Zonulae occludens
